# CD19 CAR-T cell therapy: a new dawn for autoimmune rheumatic diseases?

**DOI:** 10.3389/fimmu.2024.1502712

**Published:** 2024-12-17

**Authors:** Carlos Rangel-Peláez, Laura Martínez-Gutiérrez, María Tristán-Manzano, José Luis Callejas, Norberto Ortego-Centeno, Francisco Martín, Javier Martín

**Affiliations:** ^1^ Institute of Parasitology and Biomedicine López-Neyra, Consejo Superior de Investigaciones Científicas (CSIC), Granada, Spain; ^2^ LentiStem Biotech, Pfizer-University of Granada-Andalusian Regional Government Centre for Genomics and Oncological Research (GENYO), Granada, Spain; ^3^ Systemic Autoimmune Disease Unit, Hospital Clínico San Cecilio, Instituto de Investigación Biosanitaria Ibs, Granada, Spain; ^4^ Department of Medicine, University of Granada, Granada, Spain; ^5^ Department of Biochemistry and Molecular Biology III and Immunology, Faculty of Medicine, Instituto Biosanitario de Granada (ibs.GRANADA), University of Granada, Granada, Spain; ^6^ Department of Genomic Medicine, Pfizer-University of Granada-Andalusian Regional Government Centre for Genomics and Oncological Research (GENYO), Granada, Spain; ^7^ Instituto Biosanitario de Granada (ibs.GRANADA), University of Granada, Granada, Spain

**Keywords:** CD19 CAR-T, autoimmune rheumatic diseases, systemic lupus erythematosus, systemic sclerosis, rheumatoid arthritis

## Abstract

Autoimmune rheumatic diseases (ARDs), such as rheumatoid arthritis, systemic lupus erythematosus, and systemic sclerosis, involve dysregulated immune responses causing chronic inflammation and tissue damage. Despite advancements in clinical management, many patients do not respond to current treatments, which often show limited efficacy due to the persistence of autoreactive B cells. Chimeric antigen receptor (CAR)-T cell therapy, which has shown success in oncology for B cell malignancies, targets specific antigens and involves the adoptive transfer of genetically engineered T cells. CD19 CAR-T cells, in particular, have shown promise in depleting circulating B cells and achieving clinical remission. This review discusses the potential of CD19 CAR-T cells in ARDs, highlighting clinical achievements and addressing key considerations such as optimal target cell populations, CAR construct design, acceptable toxicities, and the potential for lasting immune reset, crucial for the safe and effective adoption of CAR-T cell therapy in autoimmune treatments.

## 1 Autoimmune rheumatic diseases. Current therapies and their limitations

Autoimmune diseases encompass a heterogeneous group of pathologies that present diverse clinical manifestations, which have a significant impact on patients’ quality of life. Although individually these diseases are rare, together they are estimated to affect 3-5% of the world population, with an increasing prevalence. The impact of autoimmune diseases is particularly concerning as they primarily affect individuals in the middle stages of life, giving rise to considerable time off work. Combined with the high cost of treatment, it results in a substantial socioeconomic burden (1). Among autoimmune disorders, rheumatic diseases primarily affect connective tissue, joints, and muscles, leading to chronic inflammation and long-term damage. The development of ARDs involves a breakdown in tolerance and immune dysregulation. This leads to an immune response against self-antigens which may be expressed in specific organs or tissues, or ubiquitously distributed. This response is mainly mediated by autoreactive T and B cells, which, along with autoantibodies, play a central role in triggering an inflammatory response and damage to multiple tissues and organs (2). Conventional treatments for ARDs rely on immunosuppressive drugs such as glucocorticoids, cyclosporine A, mycophenolate or cyclophosphamide, which dampen the immune response systemically. However, due to their lack of specificity, they can cause serious side effects and toxicity, which contribute to higher mortality and morbidity rates in these individuals (3). Advancements in treating ARDs over the past two decades arise from a deeper understanding of the immunological mechanisms involved in these diseases. Researchers have identified key immunological markers, such as cytokines and their receptors, adhesion molecules, co-stimulatory molecules or cell surface markers. This has resulted in the development of biological and synthetic drugs that target multiple pathways and components of the immune system (4).

The discovery of tumor necrosis factor (TNF-α) as a crucial cytokine in the pathogenesis of ARDs as rheumatoid arthritis (RA) led to the development of the first group of biologic drugs: TNF-α inhibitor (5). Subsequently, other immune markers associated with these diseases were identified, resulting in the development of targeted therapies against them. These markers include cytokines and their receptors (IL-1, IL-6, IL-17, IL-23), B-lymphocyte activating factor, B-cell proliferation-inducing ligand (APRIL), cell surface markers (CD20, CD52), co-stimulatory molecules (CD40/CD40-L, CD80/CD86) and signaling pathways of different cytokines such as Janus kinases (6). Due to their greater target specificity compared to conventional immunosuppressants, these therapies generally have milder side effects, with severe cases being rare (4, 6). However, these therapies do not achieve a complete remission of the disease, and relapses are common. For instance, rituximab, a B-cell depletion therapy targeting CD20, has demonstrated efficacy in the treatment of several ARDs such as RA (7) and systemic sclerosis (SSc) (8). However, it does not provide a definitive cure for the disease because it fails to eliminate B cells residing in the secondary lymphoid organs (9). The same problem applies to belimumab, which inhibits B-cell activation by targeting B-cell activating factor and is used for systemic lupus erythematosus (SLE) treatment (10). As observed, treating ARDs presents a challenge in developing strategies to restore immune tolerance and achieve complete remission. One approach involves high-dose chemotherapy combined with autologous or, in less frequent cases, allogeneic hematopoietic stem cell transplantation. However, this treatment has significant toxic effects in ARDs (11–13) representing a major challenge in managing these diseases.

Great progress has been made in cell therapy particularly due to the development of genetically modified receptors, such as chimeric antigen receptors (CARs) in T cells (CAR-T). These CAR-T therapies have shown remarkable success in removing B-cell-derived malignancies such as lymphomas and leukemias (14, 15). As a consequence, the potential of CAR-T therapies for treating certain B-cell-mediated ARDs such as SLE (16, 17) has recently been explored, offering a promising and innovative treatment modality. In addition, variations of CAR-T cells including chimeric autoantibody receptor T cells (CAAR-T) (18), targeting cells producing a specific autoantibody, or CAR regulatory T cells (CAR-T_reg_) (19), which can restore immune tolerance, are also under investigation.

The purpose of this review is to comprehensively analyze the recent advances of CAR-T cell therapy for ARDs.

## 2 Biology behind chimeric antigen receptor T

T lymphocytes are central players in the defense system of the body with an important role in adaptive immunity against pathogens and tumor cells. These lymphocytes primarily are matured in the thymus, where they acquire their antigen receptor, known as the T-cell receptor (TCR). Adoptive T-lymphocyte transfer emerged as an initial strategy based on the TCR, which consists of collecting and expanding autologous T cells from the patient capable of recognizing and eliminating tumor cells carrying specific antigens. This strategy was aimed to validate the ability of the cellular components of the immune system to reject tumors and is the basis for many gene immunotherapy strategies, including CAR-T cells (20).

CAR-T therapy involves genetically modifying T cells to express a chimeric antigen receptor (CAR), enabling them to recognize and eliminate cells expressing a particular antigen in a very potent response. The term “chimeric” comes from the diverse origins of the CAR individual components: an antibody-derived extracellular antigen recognition domain, normally from a single-chain variable fragment (scFv), a transmembrane domain and an intracellular activation domain derived from T-cell signaling molecules. For CAR-T cell generation, retroviral (either gamma-retroviral or lentiviral) vectors are commonly used for stable expression of the CAR-encoding transgene in T cells. Once modified, these cells are expanded *ex vivo* for a short period of time (~7 days) and then delivered to the patient, generally intravenously (20).

In 1989, the first synthetic receptor was designed by combining the constant region of the T-cell receptor and the variable region of an antibody (21). However, challenges in culturing T cells and the initial design (scFv-TCRα or scFv-TCRβ), limited its clinical applicability. Later designs, which incorporated the intracellular domain of CD3ζ (22) and other co-stimulatory molecule domains such as CD28 (23), substantially enhanced the potential of these chimeric receptors. Finally, advancements in CAR engineering, coupled with innovations in gene transfer systems based on retroviral vectors (24), allowed successful translation of these technologies to patients, obtaining clear benefits in the clinic, particularly when targeting CD19 (25). These results boosted both public and private investment in these strategies and today there are more than 10,000 articles and 1,700 clinical trials exploring the use of CAR-T cells against various antigens. Notably, these studies resulted in the approval in 2017 of the first gene therapy drug for treating refractory type B neoplasms (26), marking a milestone in cancer treatment. Although the COVID-19 pandemic has slowed down CAR-T therapy progress, it remains as a transformative innovation that is constantly evolving. This is reflected in the diversity of products and therapeutic targets currently under investigation, along with the approval of 5 additional drugs (27).

### 2.1 Generation of CAR-T cells

**Figure 1:**
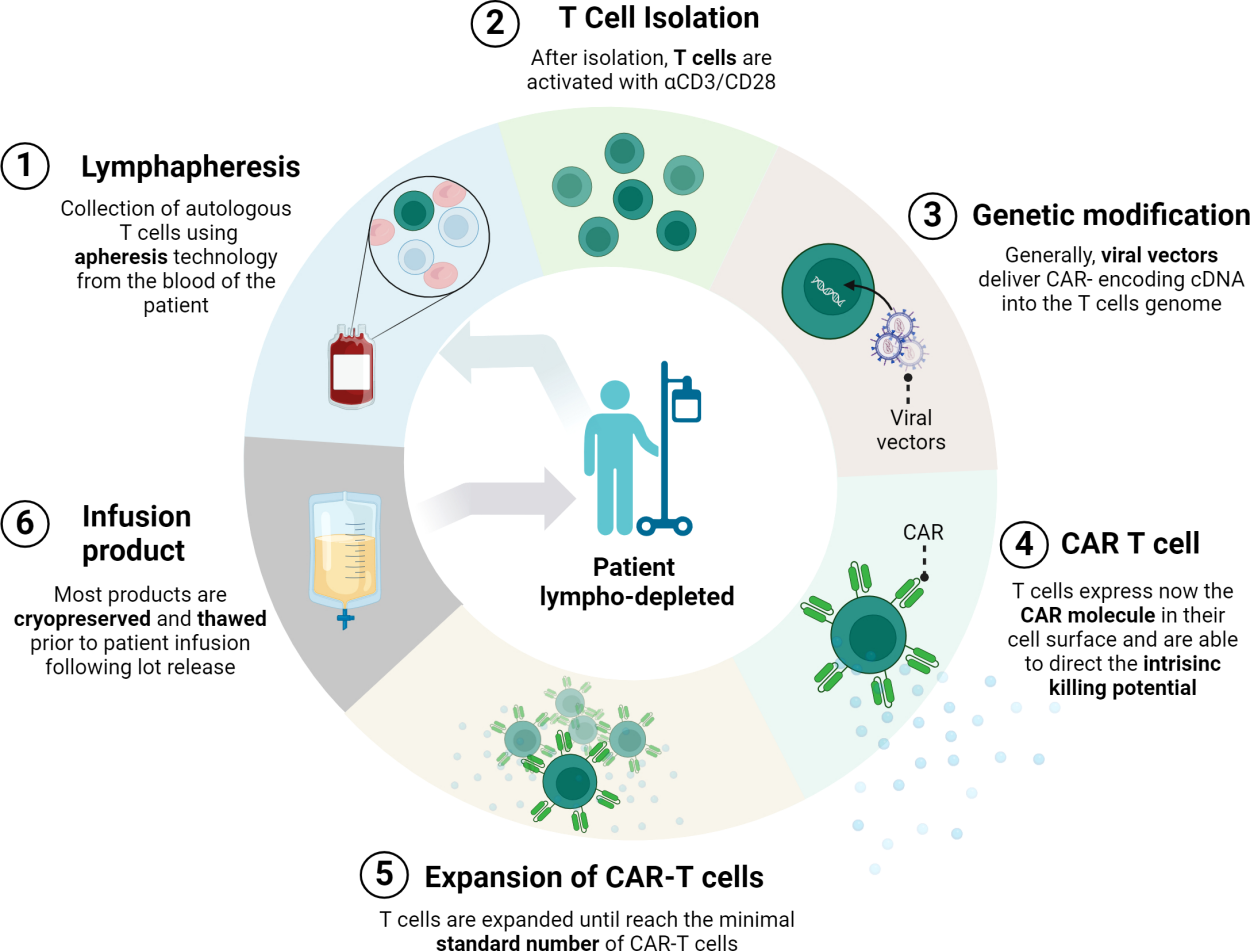
CAR-T manufacturing process. First, cells from the patient’s immune system are collected by leukapheresis. Then, T cells are separated from other blood components and undergo a gene transfer process, usually using a viral vector. This results in the expression of chimeric antigen receptors (CARs) on the surface of the T cells. After *ex vivo* amplification of the modified T cells, and after batch validation, this product re-infused into the same patient. Prior to infusion, patients normally undergo lymphocyte depletion conditioning chemotherapy. (Figure created with www.biorender.com).

In general, CAR-T cell manufacturing involves customizing the patient T cells to avoid allogeneic rejection or graft-versus-host disease (11). The process begins with the collection of T cells from the blood of the patient by apheresis. This technique selectively removes certain cells, such as T cells, while returning other remaining blood components to the patient. The collected cells are often transduced with a retroviral vector (gamma-retroviral or lentiviral) carrying the CAR construct. Subsequently, these engineered T cells are cultured and expanded *in vitro*. Prior to CAR-T cell infusion, the patient undergoes preparative lymphocyte depletion using chemotherapeutic agents in order to promote engraftment and survival of incoming CAR-T cells. Finally, CAR-T cells are infused back into the patient, where they attack cells expressing CAR-recognized markers (27) (**Figure 1**).

The conventional CAR-T manufacturing normally requires more than 10 days of *ex vivo* processing. In addition, the whole process from the apheresis to the infusion can involve a longer period of time (5-6 weeks) considering transportation, testing, product release acceptance and patient’s clinical state (28). Unfortunately, around 20–30% of patients were not infused with CAR-T cells due to rapid disease progression and death or CAR-T cell manufacturing failure, as reported recently (29, 30). Novel platforms for accelerated manufacturing are emerging, drastically reducing the manufacturing period to ~2 days, as well as production costs, probability of manufacturing failures, and vein-to-vein time (31, 32).

However, the quality of starting material varies from patient to patient and is generally lower than the potency of healthy donor cells. To drastically reduce the vein-to-vein time and costs while expanding accessibility, one promising approach is the infusion of pre-manufactured allogeneic CAR-T cells, designed as “off-the-shelf” therapies. Unlike autologous CAR-T cells, which are derived from the patient’s own cells, allogeneic CAR-T cells are sourced from healthy donors. This allows for immediate availability and scalability, as each batch generated from one donor can potentially treat up to 100 patients (33).

In a typical off-the-shelf CAR-T product, T cell receptor (TCR) and/or Major Histocompatibility Complex (MHC) genes are knocked out using gene-editing tools like TALENs or CRISPR/Cas9 (34–36). This reduces the risk of graft-versus-host disease and enhances immune compatibility, which makes allogeneic CAR-T therapies a potentially scalable option for treating various autoimmune disorders.

Recently, Wang and colleagues (33) reported the first-in-human use of allogeneic multi-edited anti-CD19 CAR-T cells (TyU19 cells) generated with CRISPR/Cas9 to knock out human leukocyte antigen (HLA)-A, HLA-B, class II major histocompatibility complex transactivator (CIITA), T cell receptor alpha constant (TRAC) and PD-1, while preserving selected MHC components to prevent natural killer cell targeting. In the clinical trial (NCT05859997), three patients with therapy-resistant autoimmune diseases—including immune-mediated necrotizing myopathy (IMNM) and diffuse cutaneous systemic sclerosis (SSc)—showed a reduction in disease markers and improved quality of life over six months, with no severe side effects such as cytokine release syndrome or graft-versus-host disease (GvHD). This study paves the way for more affordable CAR-T cell therapies for autoimmune diseases; however, longer-term results are necessary to further validate these findings.

Other recent sources of T cells for the generation of CAR-T cells are γδT cells, which represent only 0.5-5% of total T cells (37) and exhibit interesting advantages over classical αβT cells. γδT cells are capable of targeting antigen-negative tumor cells and recognizing a broader range of antigens independently of MHC presentation. Additionally, they have reduced alloreactivity which makes them promising candidates for off-the-shelf use from healthy donors (37, 38), since γδT populations are typically altered in autoimmune diseases (39). Furthermore, γδT cells naturally exhibit both cytotoxic and regulatory immune functions, which could help modulate the tumor microenvironment and reduce excessive immune responses. However, there are several challenges associated with their low frequency and their limited expansion potential. In this context, the first allogeneic gamma delta CAR T cell therapy product targeting CD20, ADI-001 (40), is currently enrolling patients with autoimmune diseases—including lupus nephritis (LN), systemic lupus erythematosus (SLE), systemic sclerosis (SSc), and anti-neutrophil cytoplasmic autoantibody (ANCA)-associated vasculitis (AAV)—for a Phase I clinical trial (NCT06375993), paving the way for γδ-CAR-T cells in treating immune-related diseases.

### 2.2 CAR-T designs

**Figure 2:**
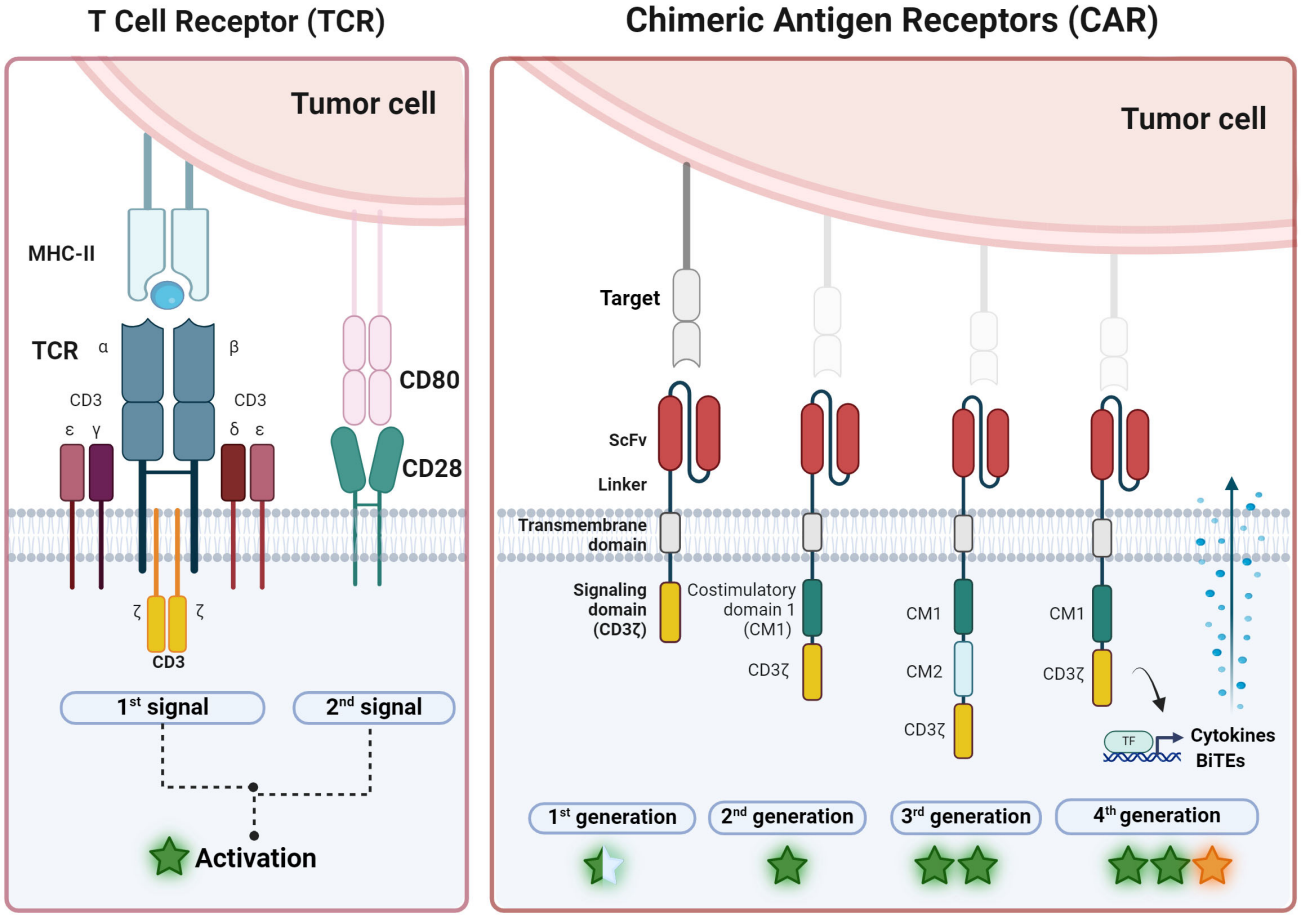
Overview of CAR-T development from TCR. (Left) T cell receptor (TCR) is a natural complex formed by six chains: two variable chains (α and β) that bind specific antigens after MHC-II presentation and four constant chains (γ, 𝛿, ϵ and 𝜁) that activate the T cell after 1^st^ signal. Second co-stimulation signal, normally from CD28, is necessary for maintaining activation. (Right) On the other hand, chimeric antigen receptor (CAR) is an artificial receptor with three domains: one that recognizes antigens independently of the HLA complex (scFv, single chain variable fragment) and is linked by a transmembrane domain to the activation domain CD3𝜁 to activate the T cell in the first generation of CAR. Second and third generation includes one and two more costimulatory domains (CM1 and CM2), respectively. Most frequent CM encloses CD28 or 4-1BB. Forth CAR generation also express bioactive molecules such as cytokines or Bi-specific T-cell engagers (BiTEs). General levels of activation or potency are represented with a star symbol: yellow star indicates the potential to modulate microenvironment (figure created with www.biorender.com).

Over the years, different CAR designs have been developed (41, 42) aiming to mimic the TCR signaling (**Figure 2**, TCR box) but directed through an antibody domain. Early designs of CARs included a scFv domain paired with a TCRα or TCRβ receptor chain (21) and then, first generation CARs, which consisted of a scFv domain bound to a transmembrane region and the CD3ζ signaling domain (22) (**Figure 2**, CAR box, left). While this construct mimicked the first TCR signal upon CAR binding to its target antigen, they lacked the second signal necessary for efficient T-cell activation. Not surprisingly, therefore, the initial CAR constructs based on the first-generation design were not sufficient for directing effective immune responses (20).

To overcome this limitation, second-generation CARs were developed (**Figure 2**, CAR box, second-left). These designs incorporate an additional intracellular motif: the signaling domain of co-stimulatory receptors such as 4-1BB (CD137) or CD28, allowing the generation of antigen-specific T cells able to expand and secrete interleukin-2 (IL-2) after repeated antigen exposures. Second-generation CARs have proven to be more effective and versatile in clinical practice, as well as more persistent in the patient, forming the basis of all CAR-T therapies approved as drugs (27). Despite these impressive results, there are still many aspects that need to be optimized (15). Relapses occur in a high percentage (between 30-80%) of patients with hematological malignancies and effectiveness is limited in myeloid leukemias and solid tumors. New CAR designs (43) and new expression methods (44) are being explored in order to tackle these challenges. For instance, modifying T cells to express other potentiating molecules, such as IL-12, IL-18, or bispecific T-cell engagers (BITEs), to increase their potency and counteract the tumor microenvironment’s immunosuppressive effects (such as fourth generation CAR-T cells or T cells redirected for universal cytokine-mediated killing (TRUCKs) (**Figure 2**, CAR box, right).

## 3 Applications of CAR-T therapy in autoimmunity

The success of CD19 CAR-T therapy in B-cell malignancies has allowed its application in patients affected by autoimmune diseases in which B cells contribute to such pathogenesis (14, 26, 45–47). It has been shown that monoclonal antibodies directed against the CD20 marker are not able to eliminate all autoreactive B cells, since this marker is not present on all B cell subtypes (13, 48). In contrast, the CD19 marker manifests in the entire B-cell lineage, including plasma cell precursors (plasmablasts). As a consequence, CAR-T strategies based on the CD19 marker have been developed. This results in the depletion of an increased number of autoreactive B cells, especially in lymphoid organs and inflamed tissues (6).

Fast expansion and activation of CAR-T cells after infusion can lead to excessive cytokine release due to signal amplification, non-specific activation of CAR-T cells, the inflammatory microenvironment, and patient-related factors. This excessive cytokine release can cause cytokine release syndrome (CRS), which is a potentially serious complication of CAR-T therapy, causing even death in some patients (grade IV). Furthermore, CAR-T therapy in autoimmune diseases has other limitations, such as increased infection susceptibility due to B cell depletion, compromising the immune system.

**Figure 3:**
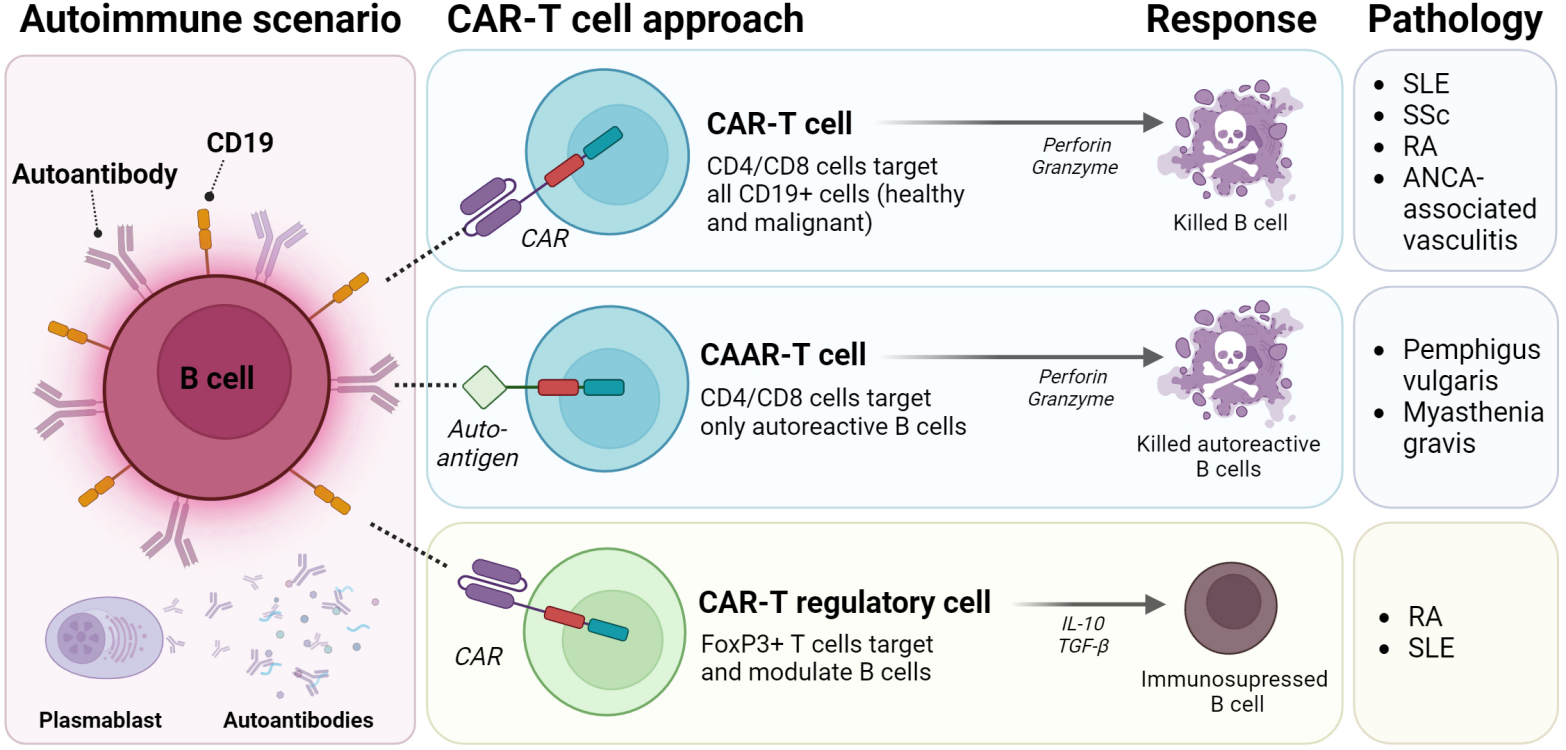
CAR-T strategies for autoimmune diseases. Classic CAR-T strategies genetically modified CD4/CD8 T cells for expressing CD19 and target healthy and autoreactive B cells, thus releasing perforin and granzyme to induce cell death. CAAR-T cells are a version of classic CAR-T cells, that expresses a Chimeric Autoantibody Receptor (CAAR) directed exclusively to autoreactive B cell clones. Third strategy modified T regulatory or T cells to express FoxP3+ and to modulate the autoreactive response. SLE: Systemic Lupus Erythematosus; SSc: Systemic Sclerosis; RA: Rheumatoid Arthritis; ANCA-associated vasculitis: Anti-Neutrophil Cytoplasmic Antibody associated vasculitis. (Figure created with www.biorender.com).

In order to selectively target autoreactive B cells and avoid eliminating all B cells from the body, novel approaches such as CAAR-T cells (Chimeric AutoAntibody Receptor) (18) are being explored. Unlike CAR-T cells which are specific for markers expressed on a certain cell type, CAAR-T cells express an extracellular autoantigen recognized by the B-cell receptor (BCR). This recognition activates CAARs, leading to specific lysis of pathogenic B cells (49) (**Figure 3**).

Additionally, CD4+ T_reg_ cells crucial for immunosuppression and tolerance, are dysregulated in autoimmune diseases (50). As a consequence, different T_reg_-based therapeutic strategies have been proposed in order to restore immune tolerance in affected tissues. Integrating CAR receptors into T_reg_ cells could enhance specificity, directing them towards antigens not necessarily presented by MHC molecules (19) (**Figure 3**).

Indeed, CAR-T cells have been shown to successfully target B cells in cancer. Therefore, it seemed logical to consider their potential use in treating autoimmune diseases, where B cells play a relevant role, such as systemic lupus erythematosus or systemic sclerosis, among others (**Table 1**).

**Table 1:** Summary of human clinical trials with CAR-T cells in rheumatic autoimmune diseases.

Disease	Treatment	Nº patients	Previous Treatment		Adverse Effects Grade after CAR-T infusion (Nº Patients | Average Recovery Time)		Follow-up time (months)	Refs.
			Non-Biological	Biological	CRS	ICANS		
SLE	CD19 CAR-T Cells	8	✓	✓	1 (5 | 5 days)	0	6 - 29	(47)
SLE	CD19 CAR-T Cells	1	✓	✓	1 (1 | 4 days)	0	6	(82)
SLE-ITP	CD19 CAR-T Cells	1	✓	✓	1 (1 |No data)	0	6	(57)
SLE-LN	BCMA/CD19 CAR-T Cells	11	✓	✓ (7) ✘ (4)	1 (9 | No data)	0	6-12	(59)
SLE	BCMA/CD19 CAR-T Cells	2	✓	✘	0	0	12-24	(59)
SSc	CD19 CAR-T Cells	4	✓	✓ (1) ✘ (3)	1 (3 | 5 days)	0	4; 7; 10; 13	(47, 62)
SSc	CD19 CAR-T Cells	1	✓	✘	1 (1 | 5 days)	0	11	(63)
RA	CD19 CAR-T Cells	1	✓	✓	1 (1 | No data)	0	5	(64)
IIM	CD19 CAR-T Cells	3	✓	✓	1 (2* | 5 days); 2 (1 | 5 days)	1 (1* | 14 days)	5; 18; 18	(47, 83, 84)
IIM	CD19 CAR-T Cells	1	✓	✓	1 (1 | No data)	0	8	(85)
MS	CD19 CAR-T Cells	2	✓	✓	1 (1 | ~5 weeks)	0	~1; ~3	(68)
SjS	CD19 CAR-T Cells	1	✓	✓	2 (1 | 3 days)**	1 (1 | 11 days)	6	(86)

CRS, Cytokine Release Syndrome; ICANS, Immune Effector Cell-Associated Neurotoxicity Syndrome; SLE, Systemic Lupus Erythematosus; ITP, Immune Thrombocytopenia; LN, Lupus Nephritis; SSc, Systemic Sclerosis; IIM, Idiopathic Inflammatory Myopathies; MS, Multiple Sclerosis; SjS, Sjögren Syndrome; Refs, References. * Same patient. ** Immediately before CRS grade II, this patient suffered CRS grade I for 5 days.

### 3.1 CAR-T in systemic lupus erythematosus (SLE)

Systemic lupus erythematosus (SLE) is characterized by the production of autoantibodies against nucleic acids or DNA-binding proteins, leading to immunocomplexes deposition in tissues, promoting tissue damage. Currently, there is no cure for SLE and existing treatments such as glucocorticoids, antimalarials and monoclonal antibodies such as belimumab (blocks the B-lymphocyte stimulating factor BLyS/BAFF) or anifrolumab (inhibits the interferon type 1 receptor -IFNAR1-) are insufficient for controlling the disease. Despite B cells playing a key role in the SLE pathogenesis, B cell depletion with current monoclonal antibodies such as belimumab fails to achieve symptom remission in many lupus patients (10).

Initially, studies in murine models of SLE showed efficacy of anti-CD19 CAR-T therapy in B-cell depletion and reduction of disease progression (51, 52). Subsequently, this therapy was tested in a patient with severe and refractory SLE who had not responded to other treatments (17). The short-term results (44 days) showed a decreased disease activity, as evidenced by a decrease in the Systemic Lupus Erythematosus Disease Activity Index (SLEDAI) and disappearance of SLE autoantibodies, in contrast to anti-CD20 monoclonal antibodies. This suggests that plasmablasts are the major source of autoantibodies. In fact, a recent study investigating lymph nodes in patients receiving CD19 CAR-T therapy revealed a remarkable complete depletion of B cells in 6 SLE and 2 SSc patients, a level unattainable with monoclonal antibodies like rituximab (53). Importantly, the patient did not experience adverse effects such as cytokine release syndrome (CRS), neurotoxic effects or prolonged cytopenia. The reduced CRS and neuroinflammation side effect on lupus and other autoimmune patients treated with CAR-T cells was further confirmed in other clinical trials (47, 54–56). The lower CRS in patients with autoimmune disorders is somehow surprising, since SLE poses a higher risk of macrophage activation syndrome. Still, the main hypothesis behind the lower CRS observed with CAR T cell therapy in autoimmune patients is the lower B cell burden compared to those found in B cell malignancies. Similarly, the lack of neurotoxicity in autoimmune disease´s patients treated with CAR-T is not fully understood. However, the current safety data on CAR T cell treatment of Lupus and other autoimmune diseases need to be interpreted with caution as only a limited number of patients have been treated to date. Another study involving five patients, not only obtained similar results in terms of efficacy and safety, but also suggested that this therapy could lead to drug-free remission of the disease (16).

Long-term follow-up of 8 SLE patients, including the previously mentioned patient, demonstrated significant progress with CD19 CAR-T therapy. Specifically, the follow-up period ranged from 6 to 29 months after CAR-T cell infusion. In the short term, CAR-T therapy demonstrated its efficacy, while long-term observations showed symptom remission in all patients, despite inadequate responses to other immunosuppressive treatments. Moreover, the B-cell population reconstituted itself 2 years after treatment, without relapse symptoms. On the other hand, grade I CRS occurred in only 5 of 8 patients, characterized by mild fever and a mean recovery time of 5 days, with no major complications. Only one patient experienced an infection that entailed hospitalization, although it was managed with the administration of antibiotics. Thus, it was demonstrated that a single CD19 CAR-T infusion can lead to long-term, drug-free SLE remission (47).

Moreover, given the preliminary data’s insufficiency in SLE associated malignancies, a recent case report (57) suggests that CD19 CAR-T therapy may hold promise for SLE patients with immune thrombocytopenia (ITP). A patient with SLE-ITP underwent CD19 CAR-T treatment, resulting in significant B cell depletion. While B cell recovery was observed, it did not fully restore pre-treatment levels within six months. Although this case report offers initial insights, further research is needed to establish the safety, efficacy, and optimal dosing of CD19 CAR-T therapy for SLE-ITP.

The use of dual-targeting strategies was explored by combining CD19 with additional surface markers, such as BCMA (B-cell maturation antigen), with the aim of enhancing efficacy and reducing the risk of relapses. BCMA is expressed by long-lived plasma cells, which are implicated in the pathogenesis of SLE and lupus nephritis (LN) (58). A recent phase 1 clinical trial (59) evaluated the safety and efficacy of BCMA-CD19 compound CAR (cCAR) therapy in patients with SLE-LN. Eleven patients aged 17-46 participated in the study. The cCAR therapy led to a significant reduction in SLEDAI score and improved renal function. Additionally, a patient with SLE-ITP experienced resolution of thrombocytopenia and achieved medication-free remission. B cell populations recovered within 2-6 months post-cCAR in most patients, with no indications of SLE relapse. While grade I CRS occurred in nine patients, no ICANS were reported.

Despite their demonstrated efficacy, CAR-T cell therapy carries an increased risk of infections and side effects. Therefore after the identification of induced autoantigens in SLE such as nuclear antigens, cytoplasmic antigens, cell membrane antigens, phospholipid-related antigens, blood cells, endothelial cells and nervous system antigens (60), the design of CAAR-T targeting B cells specific to these autoantigens has been proposed (49) (**Figure 3**).

In addition to the functional impairment of B cells, in SLE there is also a reduction in the number and function of T_reg_ cells (10). As a consequence, anti-CD19 CAR-T_reg_ overexpressing FoxP3 has been developed (**Figure 3**). These CAR-T_reg_ cells have shown promise in alleviating SLE symptoms by exhibiting immunosuppressive activity in B cell activation *in vitro* and reducing autoantibody levels in mouse models (61).

### 3.2 CAR-T in systemic sclerosis

Systemic sclerosis (SSc) is a rare autoimmune disease affecting connective tissue. It is characterized by immune system dysregulation followed by vascular damage and fibrosis. This is classified based on the extent of fibrosis into a) limited cutaneous SSc (lcSS) involving fibrosis of the skin distal to the elbows and/or knees, but without involvement of the trunk, although skin thickening may occur on the face and neck; and b) diffuse cutaneous SSc (dcSS), which affects the skin both distal and proximal to the knees and/or elbows and/or trunk (12). The vast majority of patients with SSc present Raynaud phenomenon and involvement of organs such as the skin and lung. Serologically, patients may present anti-RNA polymerase III (anti-RNAPIII), anti-centromere (ACA) or anti-DNA topoisomerase (Scl70+) autoantibodies (12).

Standard treatment for SSc includes immunosuppressants such as mycophenolate mofetil, methotrexate, cyclophosphamide. When resistance to these drugs occurs, other alternatives are considered. For instance, tocilizumab (which blocks the IL-6 receptor) or nintedanib (a tyrosine kinase receptor inhibitor) may be used in cases of pulmonary fibrosis. Rituximab is also employed because it targets B cells, which play a crucial role in the disease. However, similar to SLE, CD20+ B cell depletion is not always sufficient to alleviate the symptoms of the disease, since it does not eliminate expanded B cell precursors, nor the plasmablasts, which are possibly responsible for the autoantibodies production (12). Autologous hematopoietic stem cell transplantation is only indicated for severe cases of SSc with an unfavorable prognosis, since the mortality associated with this therapy is high (62).

Several studies have demonstrated the effectiveness of CAR-T therapy in SSc. In a notable case, a 60-year-old patient with diffuse SSc, who presented complications such as pulmonary fibrosis and hypertension and myocardial fibrosis, as well as high levels of anti-RNAPIII antibodies, received CAR-T therapy. The treatment was well-tolerated, resulting in mild fever (grade I CRS) and no signs of immune effector cell-associated neurotoxicity syndrome (ICANS). A reduction in RP11 autoantibody levels was observed, along with improvement in pulmonary and myocardial fibrosis. In addition, the EUSTAR (European Scleroderma Trials and Research Group) Activity Index, which measures disease activity and severity based on different clinical parameters, decreased, indicating an improvement in disease activity. The patient also reported a reduction in Raynaud phenomenon symptoms (62).

In another case study, a patient with Scl70+ SSc and nonspecific interstitial pneumonia (NSIP) received third-generation CD19-CAR-T (63). Remarkably, no CRS symptoms were observed. The patient demonstrated an improvement in pulmonary function and regression of skin fibrosis, as assessed by the Modified Rodnan Skin Score (mRSS). In addition, CAR-T cells persisted for 11 months, during which autoantibodies decreased. Also noteworthy was the disappearance of immunocomplexes activating the Fcɣ receptor, which contribute to the pathology of SSc Scl70+ (63). These two clinical cases provide promising evidence for the short-term efficacy of CAR-T therapy in treating SSc.

Recently, a short-term follow-up study was conducted on two SSc patients who received allogeneic CD19 CAR-T therapy (33). Both patients experienced temporary B cell depletion, followed by recovery beginning at month two and reaching basal levels by month six. Interestingly, a decrease in disease activity, as measured by mRSS, was reported. Additionally, interstitial lung disease severity was markedly reduced in both patients, as evidenced by decreased bilateral pulmonary inflammation, interlobar effusion, and fibrous strands. Furthermore, a significant decrease in anti-Scl-70 autoantibody levels in both patients was observed. Importantly, neither patient developed CRS or ICANS. These reported findings offer a promising treatment option for SSc (33).

Long-term follow-up of 4 SSc patients, including the aforementioned 60-year-old patient, demonstrates the efficacy of CD19 CAR-T therapy. The follow-up period ranged from 4 to 13 months after CAR-T cell infusion. In all patients, overall disease activity decreased, as well as the EUSTAR index and mRSS. Moreover, disease-specific autoantibodies (anti-Scl70, anti-Th/To, anti-PM-Scl70) were also reduced 6 months after treatment. By the end of the follow-up, recovery of the B-cell population was observed, allowing the discontinuation of glucocorticoids and immunosuppressive medications. Regarding adverse effects, 3 of the 4 patients suffered grade I CRS that recovered within 5 days. In conclusion, the single infusion of CD19 CAR-T was effective in achieving long-term remission of the disease and without additional drug requirements (47).

### 3.3 CAR-T in rheumatoid arthritis

Rheumatoid arthritis is a chronic autoimmune disease that primarily affects the joints and periarticular soft tissues. The mainstay of RA treatment is disease-modifying antirheumatic drugs (DMARDs), particularly methotrexate, which are used to prevent joint degradation. Biological treatments, especially those targeting TNF (e.g., adalimumab, infliximab, or etanercept), are also commonly employed. Short-term corticosteroid use may be considered to manage inflammation when initiating or adjusting DMARD therapy, but it is recommended at the lowest effective dose and for the shortest duration possible. Non-steroidal anti-inflammatory drugs (NSAIDs), such as naproxen, are also used for symptomatic relief (3).

In RA, B cells accumulate in the synovial cavity, where they generate autoantibodies that contribute to joint degradation. These autoantibodies include rheumatoid factor (RF) and anti-citrullinated peptide antibodies (ACPA) (3). A recent study demonstrated that an ACPA-positive RA patient with AChR-antibody-positive myasthenia gravis responded positively to CD19 CAR-T therapy. This treatment led to an improvement in disease activity, as indicated by the Disease Activity Score-28 based on erythrocyte sedimentation rate (DAS-28-ESR), Disease Activity Score-28 based on C-reactive protein (DAS-28-CRP), and Clinical Disease Activity Index (CDAI), scores indicating remission. Interestingly, it resulted in a decrease in ACPA levels but not in anti-AChR antibody levels, suggesting that CD19+ plasmablasts may not be the primary source. Despite that, the activity index of myasthenia gravis decreased, as assessed by the quantitative myasthenia gravis score (QMG-score) and myasthenia gravis activities of daily living (MG-ADL). This implies that there may not be a direct correlation between autoantibody levels and the clinical activity of myasthenia gravis. Only a mild grade I CRS was observed during the 150 days-follow up, indicating that the treatment was well tolerated (64).

Due to the presence of ACPA in RA, the use of CAR-T specific for ACPA-producing B cells has been proposed. Specifically, a strategy has been developed based on the generation of CAR-T cells modified to recognize fluorescein isothiocyanate (FITC)-labeled citrullinated peptides. These peptides, added exogenously, are also recognized by the receptor of these autoantigen-specific B cells. In this way, a specific interaction is established between autoreactive B cells and CAR-T, allowing the selective elimination of various types of autoreactive B cells that recognize citrullinated autoantigens, such as vimentin, type II collagen, fibrinogen and tenascin-C13 (65).

Another promising approach involves the induction of tolerance in the synovial cavity of joints by CAR-T_reg_ cells. Preliminary studies suggest that T_reg_ cells modified to recognize citrulline vimentin (CV), found exclusively in the extracellular matrix of inflamed synovial tissue, could attract CAR-T_reg_ cells to the site of inflammation (49).

### 3.4 CAR-T in anti-neutrophil cytoplasmic antibody-associated vasculitis

Anti-neutrophil cytoplasmic antibody (ANCA)-associated vasculitis is group of autoimmune diseases that affect small and medium-sized blood vessels and are characterized by the production of ANCA directed against proteinase 3 (PR3-ANCA) or myeloperoxidase (MPO-ANCA) by B cells. These antibodies activate myeloid cells, especially neutrophils and monocytes that trigger vascular inflammation and tissue damage (66).

This type of vasculitis is classified in three types according to clinical features: granulomatosis with polyangiitis, microscopic polyangiitis and eosinophilic granulomatosis with polyangiitis. Its clinical presentation is usually heterogeneous, with the respiratory tract and the kidney being the most frequently affected organs. In this regard, glomerulonephritis represents one of the most serious clinical complications. Combination of non-biological drugs such as glucocorticoids and azathioprine with rituximab is the first-line treatment for ANCA patients (66).

Recent studies in mouse models of MPO-ANCA vasculitis have demonstrated the efficacy of CD19 CAR-T therapy. This approach led to a significant reduction in B cells and plasmablasts providing protection against renal tissue damage (67). These findings suggest that CAR-T therapy may offer a therapeutic alternative for ANCA vasculitis patients who do not respond adequately to current treatments. However, further studies are needed to validate its efficacy in humans.

### 3.5 CAR-T for other autoimmune diseases

The potential of CAR-T therapy extends beyond ARDs (46). In multiple sclerosis, CD19 CAR-T cells have demonstrated success in reducing autoreactive B cells and IgG levels within the central nervous system, leading to improved symptoms in patients (68). Similarly, refractory myasthenia gravis patients have shown positive responses to CAR-T therapy targeting BCMA and CD19, suggesting a potential breakthrough for this challenging condition (69, 70). Preclinical studies have also demonstrated the efficacy of CAR-T cells designed to target antigen-presenting cells responsible for activating autoimmune T cells in type 1 diabetes (71). Furthermore, CAAR-T cells targeting specific B cells in myasthenia gravis (72) and pemphigus vulgaris (18) have also shown efficacy and safety in preclinical studies.

## 4 Limitations and future prospects

CAR-T therapy represents a potential alternative for patients who do not respond to traditional treatments. However, it presents several limitations.

First of all, only one long-term study (29 months) has been performed, showing that the CAR-T cell residence time in the individual is relatively short (between 26 and 79 days). Moreover, previous immunosuppressive treatments further compromise the duration of CAR-T cells. In that study, patients treated with rituximab had less CAR-T cell persistence than those without rituximab treatment. Despite this, the short-term depletion of autoreactive cells may be sufficient, as no signs of the diseases were observed during the duration of the study. However, studies evaluating patients over a longer period of time are required to test whether further persistence of CAR-T cells is needed in the individual (11, 13). A possible solution would be the use of third-generation CAR-T cells, which may enhance effectiveness and durability (73).

In some cases, B-cell elimination alone is not enough to control the disease due to long-lived plasma cells persistence in the bone marrow, which may contribute to the production of autoantibodies and do not express the CD19 marker. Therefore, developing CAR-T cells targeting other plasma cell-specific markers such as CD38 or BCMA becomes particularly relevant (74–76). In addition, clinical symptoms in autoimmune diseases are often irreversible, such as renal damage in SLE or pulmonary fibrosis in systemic sclerosis, thus preventing effective treatment with CAR-T (13, 54). Therefore, early diagnosis and rapid treatment are essential to minimize the risk of permanent organ damage.

On the other hand, like conventional therapies, CAR-T therapy also leads to immunosuppression, increasing the risk of infections. In addition, it presents some adverse effects including fever or fatigue or even serious complications such as cytokine release syndrome or neurotoxicity. Cytokine release syndrome (CRS) is less frequent in patients with autoimmunity than in patients with cancer, due to lower B-cell burden in autoimmune diseases (77). Treatment to prevent CRS and neurotoxicity is based on antipyretics, glucocorticoids and IL-6 receptor blockade with tocilizumab. However, the use of glucocorticoids or other treatments to prevent CRS could suppress the immune system, which may lead to a reduction in the efficacy of CAR-T (78).

Finally, due to the high price of CAR-T therapy ($373,000-530,000) (79), not including hospital fees and drugs for conditioning of patients prior to administration or to treat side effects, its reach is limited to a small number of patients. However, its potential to reduce the need for long-term immunosuppressants, together with the possibility of achieving durable drug-free remission, could make it a potentially cost-effective alternative to the cost of conventional therapies ($100,000/year) (13). Similarly, the production process needs to be optimized to reduce the cost and increase the accessibility of the treatment.

## 5 Conclusion

CAR-T therapy has been successfully introduced in the treatment of autoimmune diseases. This unique approach is based on the complex manufacture of a genetically modified and personalized autologous cellular product that requires less use of conventional drugs. While the use of chemotherapy for patient´s conditioning might improve the short-term outcomes of CAR-T cell treatment, it is unlikely to achieve complete B-cell depletion, eliminate autoantibodies, or induce sustained drug-free remission. Many patients received higher doses (>5 g) of chemotherapy before CAR-T cell therapy without clinical effects (47, 80, 81). This marks the beginning of a new era in the treatment of autoimmune diseases, transforming the current principle of long-term immunosuppression into a strategy that induces an immune reboot. Importantly, research on CAR-T cell therapy for autoimmune diseases is still ongoing, and further studies are needed to confirm its long-term efficacy and safety.
